# Facilitation of dragonfly target-detecting neurons by slow moving features on continuous paths

**DOI:** 10.3389/fncir.2012.00079

**Published:** 2012-10-29

**Authors:** James R. Dunbier, Steven D. Wiederman, Patrick A. Shoemaker, David C. O'Carroll

**Affiliations:** ^1^Adelaide Centre for Neuroscience Research, School of Medical Sciences, The University of AdelaideAdelaide, SA, Australia; ^2^Tanner Research Inc.Monrovia, CA, USA

**Keywords:** salient feature, EMD, motion detection, insect vision, target tracking, feature detector, second order motion

## Abstract

Dragonflies detect and pursue targets such as other insects for feeding and conspecific interaction. They have a class of neurons highly specialized for this task in their lobula, the “small target motion detecting” (STMD) neurons. One such neuron, CSTMD1, reaches maximum response slowly over hundreds of milliseconds of target motion. Recording the intracellular response from CSTMD1 and a second neuron in this system, BSTMD1, we determined that for the neurons to reach maximum response levels, target motion must produce sequential local activation of elementary motion detecting elements. This facilitation effect is most pronounced when targets move at velocities slower than what was previously thought to be optimal. It is completely disrupted if targets are instantaneously displaced a few degrees from their current location. Additionally, we utilize a simple computational model to discount the parsimonious hypothesis that CSTMD1's slow build-up to maximum response is due to it incorporating a sluggish neural delay filter. Whilst the observed facilitation may be too slow to play a role in prey pursuit flights, which are typically rapidly resolved, we hypothesize that it helps maintain elevated sensitivity during prolonged, aerobatically intricate conspecific pursuits. Since the effect seems to be localized, it most likely enhances the relative salience of the most recently “seen” locations during such pursuit flights.

## Introduction

Detecting and tracking small moving targets within a visual scene is a complex task, yet one of great importance to many animals that have evolved sophisticated anatomical, behavioral and neural mechanisms for target analysis (Zeil, 1993; Land and Collett, 1974; Collett and Land, 1978; Wehrhahn et al., 1982; Land, 1993; Frye and Dickinson, 2007; Olberg et al., 2007). Flying insects such as the larger dipteran flies and dragonflies display a spectacular ability to track and intercept prey or conspecifics that move against visually cluttered backgrounds (Collett and Land, 1975; Nordström et al., 2009; Nordström and O'Carroll, 2009). Dragonflies capture prey with success rates of 97% (Olberg et al., 2000) yet do so even in the presence of distracters such as conspecifics or swarms of prey (Corbet, 1999).

Target selective descending neurons (TSDNs) likely to be involved in this impressive behavior were first described from the dragonfly ventral nerve cord (Olberg, 1986). Optic lobe interneurons that are inputs to such pre-motor pathways were more recently characterized in both dragonflies (O'Carroll, 1993; Geurten et al., 2007) and hoverflies (Nordström et al., 2009; Barnett et al., 2007; Nordström and O'Carroll, 2009). These “small target motion detector” (STMD) neurons display an impressive selectivity for small moving objects, yet give very robust responses even against complex backgrounds (Nordström et al., 2009; Nordström and O'Carroll, 2009).

An interesting problem that STMD neurons must deal with is that the tiny stimuli they respond to only occupy the receptive fields of photoreceptors in one or two adjacent ommatidia at a given moment. By contrast, the neurons involved in insect optic flow analysis, lobula plate tangential cells (LPTCs), can sum local motion across large arrays of detectors (Krapp and Hengstenberg, 1996). This allows them to generate a reliable global motion response to wide-field stimuli down to very low stimulus contrasts (below 3%)—extraordinary contrast sensitivity that aids coding of a wide range of image speeds (O'Carroll et al., 1996; Harris et al., 2000; Straw et al., 2006). This spatial integration also allows LPTCs to smooth out local variance (pattern noise) due to the inhomogeneous structure of the surrounding scene, further improving motion coding (Borst et al., 1995; Barnett et al., 2010; Meyer et al., 2011; O'Carroll et al., 2011). STMD neurons do not have this luxury: Some certainly have receptive fields as large as their LPTC counterparts, presumably via summation across arrays of putative local “elementary small target motion detectors” (ESTMDs) (Geurten et al., 2007; Wiederman et al., 2008). However, given the spatially circumscribed nature of the stimulus, simple spatial summation cannot improve reliability for target discrimination by averaging out local noise as the feature moves across different parts of the background.

How then does the STMD pathway respond so robustly and selectively to moving targets that occupy only a fraction of their receptive field? An interesting hypothesis emerges from our recent analysis of the response time course in the large-field dragonfly STMD neuron CSTMD1 (Nordström et al., 2011). Despite a short initial latency, CSTMD1's response to continuous target motion builds to its maximum over several hundred milliseconds. Nordström et al. (2011) hypothesized that the STMD pathway might utilize a second-order motion detector network (Zanker, 1994). This might enhance target detection by some form of additional non-linear integration of adjacent ESTMD outputs, such as the delay and correlate mechanism intrinsic to direction selective motion detectors. Such a mechanism could take advantage of a distinguishing characteristic feature of *natural* target motion: true targets tend to move along continuous paths, even if they change direction or vary in contrast as they move across the background. A response in one local motion detector ought to be well correlated with an appropriately delayed response in neighboring detectors (i.e., matching the target velocity). Noise, on the other hand (including spurious feature motion of the background caused by events such as foliage moving with wind), would be local and variable and thus less likely to persist along continuous trajectories. This second-order system would thus allow rejection of feature motion not correlated across multiple local adjacent input detectors, permitting amplification to enhance robustness whilst maintaining selectivity to stimuli on the spatial scale of single ommatidia of the eye.

In this paper we test this hypothesis by determining if the facilitation identified by Nordström et al. (2011) is propagated globally by motion throughout CSTMD1's receptive field, or requires sequential local activation (i.e., continuous motion along a trajectory). Our findings strongly support a higher-order integration mechanism, since disruption of stimulus trajectories into discontinuous paths dramatically reduces the effectiveness of stimuli that sweep the same total area of the receptive field. We also combine computational modeling with further analysis of the velocity tuning and time course of the CSTMD1 response, to rule out a parsimonious explanation that the slow facilitation time course simply reflects long delay time-constants in the underlying motion detectors.

## Materials and methods

### Electrophysiological methods

Experiments were carried out on 12 male, wild-caught dragonflies (*Hemicordulia tau*). The dragonflies were immobilized with a wax-rosin (1:1) mixture, and the head was tilted forward to gain access to the posterior head surface. A small hole was cut over the left lobula. Neurons were recorded intracellularly using aluminium silicate micropipettes pulled on a Sutter Instruments P-97 puller and filled with 2 M KCl. Electrodes typically had a tip resistance between 60 and 110 MΩ. We identified CSTMD1 by its characteristic large, biphasic action potentials and distinctive receptive field shape in the frontal dorsal visual field, mapped with a drifting target stimulus as described by Geurten et al. (2007). Visual stimuli were presented to the animals on a high resolution LCD computer monitor (Samsung 2233RZ) at 120 Hz frame rate, using VisionEgg software (Straw, 2008). The animal was placed on an adjustable stand and aligned at a fixed distance from the display, using a calibration frame fitted to the front of the display. The small access hole allowed visualization of surface landmarks on the brain only over a very limited range of angles, such that individual dragonflies were always oriented in similar positions, with the frontal midline corresponding to the horizontal center of the monitor lower edge, such that the screen center was approximately 40° above the horizon. Small individual differences in elevation alignment were further accounted for by measuring the angle of inclination of the dragonfly's head relative to the vertical. Azimuth alignment with the mid-point on the screen was subsequently confirmed by scanning the receptive field with horizontally drifted targets, since the CSTMD1 receptive field cuts off sharply at the frontal midline (see Geurten et al., 2007 and Figure 1 of Bolzon et al., 2009). The display subtended approximately 110 × 82° (width by height) at the animal's eye, with a resolution of 1680 × 1050 pixels (corresponding to 10 pixels/° at the screen center) and a background luminance of 280 Cd.m^−2^. Data were digitized at 5 kHz using a 16-bit A/D converter (National Instruments, Austin, TX, USA) and analyzed off-line with MATLAB (www.mathworks.com).

### Stimuli

**Figure 1:**
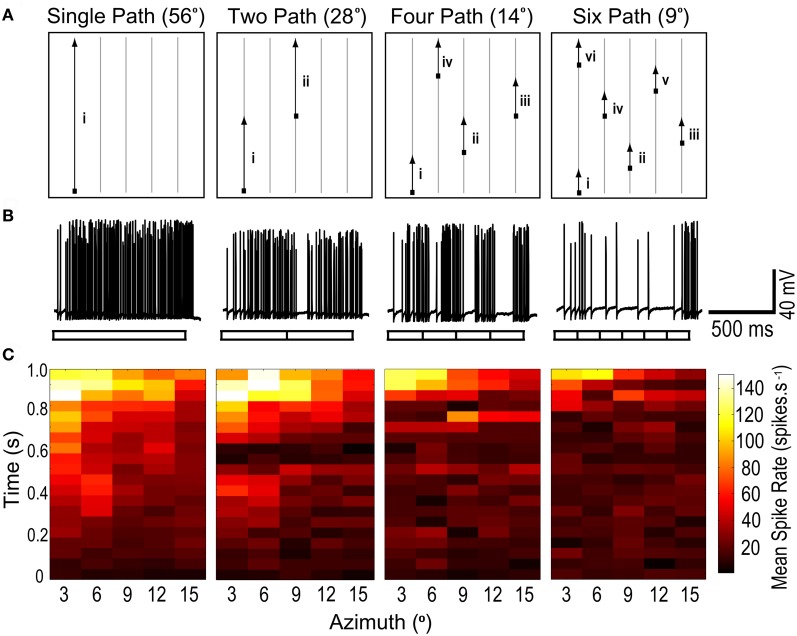
**(A)** A moving target with its path segmented into varying lengths, is presented within a region of interest (ROI) of CSTMD1's receptive field. For the single path variant, the target moves vertically to the top of the ROI. For two paths, the target traverses midway before jumping to a new horizontal location. Similarly, shorter segment lengths result in multiple jumps. A stimulus set is composed of five such traversals, each started from the bottom of the ROI, at varying horizontal locations. Thus for any segment length variant, an individual path is traversed only once and the entire ROI is covered. **(B)** Raw responses to 1 of the 5 traversals, for each of the 4 segment length variants (56°, 28°, 14°, and 9°). The neuronal response to a single continuous path (i) builds to a strongly facilitated state. In the second raw trace (two paths), CSTMD1's response resets at the single spatial discontinuity (i–ii), with responses slowly re-building to their facilitated level. Additional discontinuities reset CSTMD1's response more often thus decreasing activity as segment length decreases. **(C)** From the five traversals, responses during a segment are excised and concatenated corresponding to the spatial location of the target. These reconstructed receptive fields are then averaged (*n* = 7 neurons). From left to right, as segment length is decreased, the receptive field shows a decrease in neuronal activity across the entire ROI.

**Figure 2:**
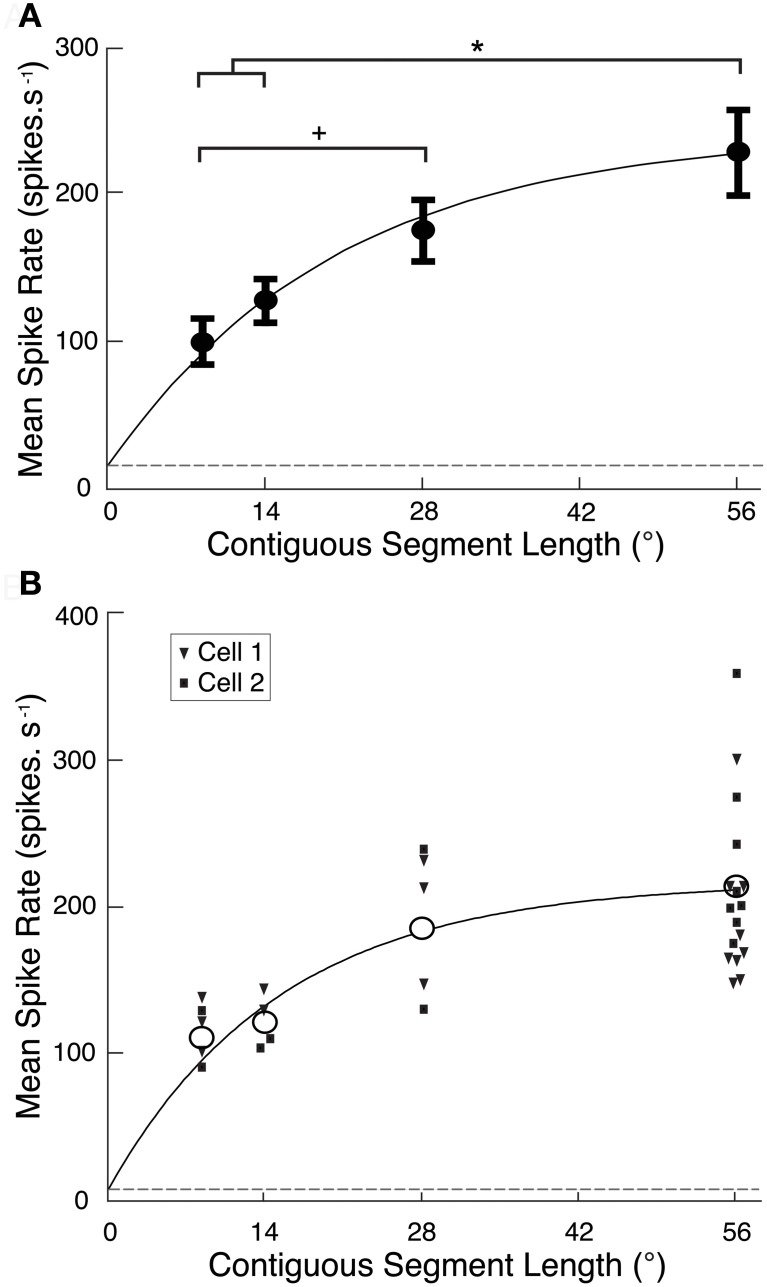
**(A)** CSTMD1 spike rate averaged over each reconstructed ROI (Figure 1C) from the segment length variants (mean ± SEM, *n* = 6 neurons). As segment length increases, overall activity increases at a decreasing rate (grey line, exponential curve fit). Though the target crosses many ommatidia at all segment lengths, the 56° single path produces significantly stronger CSTMD1 activity than either the 14 or 9° segment lengths (^*^P < 0.05). Additionally, responses to the 28° two paths is significantly higher than the 9° six paths (^+^*P* < 0.05). The dashed line indicates levels of spontaneous activity. **(B)** The mean spike rate averaged over the reconstructed ROI for each of the segment length variants in the newly described neuron, BSTMD1 (unfilled circles, mean, *n* = 2). Individual replicates of each segment length from each cell are represented as filled squares and triangles, respectively. Neuronal activity increases as segment length increases, in a similar manner to CSTMD1 (Figure 2A).

We defined a sub-region of the CSTMD1's receptive field, 56° vertical and 15° horizontal extent, in which we tested the response to long trajectories for a moving target. This region of interest (ROI) was close to the central excitatory region, but terminated slightly (4–5°) below the center of the prominent receptive field hotspot (a sub-region of the receptive field with a higher spiking response to targets) and just inside the medial boundary corresponding to the midline separating the visual hemifields (0° azimuth). Five vertical paths (56° total height, spaced at 3° intervals) were defined within this ROI (Figure 1). During each trial, a small target (~1.1° square) drifted upwards from the bottom of the trial region to the top. Targets either moved along the full 56° length of one of the five paths (single segment, Figure 2A) or “jumped” laterally to shorter segments of other paths, with either 2, 4, or 6 segments per traversal (28°, 14°, and 9° segment length respectively, Figure 1A). Each vertical traversal took the same amount of time, regardless of how many segments it was broken into. A set of five such trials was completed during each experimental run. The order in which segments were traversed ensured that each segment in a sequence was separated by at least 6° laterally (twice the path separation) from the last segment travelled, so that directly neighboring paths were never travelled consecutively (see Figure 1A). For each of the four different segment lengths, five such traversals were completed during each stimulus set, such that the entire length of each of the five paths was traversed only once. Hence, the complete stimulus set effectively measures the response from the same parts of the receptive field, for each segment length.

### Computational modelling

We simulated a one-dimensional linear array of correlation-type Elementary Motion Detectors (EMDs) in Matlab. Input stimuli were defined as animations of dark targets (nominal luminance 0) moving against a bright background (nominal luminance 1.0) with a temporal sample rate of 1000 Hz. Two input sensors [S1 and S2, sensor separation (Δ ϕ) 1°] received a Gaussian blurred (Full width at half maximum, Δ ρ 1.4°) luminance signal to account for the optics of the ommatidia. This was a wider Δ ϕ than the minimum separation measured in the dragonfly acute zone (Horridge, 1978), but it was more representative of the average Δ ϕ across CSTMD1's large receptive field, as traversed by our target stimuli. Each signal arm was convolved with a lognormal temporal filter that mimics the temporal low-pass properties of the insect photoreceptor (T1) (*T*_p_ = 10.1 ms, σ = 0.197). The output of S1 was then further convolved with a linear first-order delay filter (TD) with a variable time constant. This delayed signal was multiplied with the undelayed signal from S2. This process was repeated in a mirror symmetrical fashion and the two outputs subtracted from each other to give an individual EMD's response. Since discrete features stimulate individual EMDs at different times relative to stimulus onset, model outputs were then summed linearly across an EMD array limited in size (for computational efficiency) to that traversed at the velocity of a given stimulus. Simulations were run at a sample rate of 1 KHz and 1000 samples per degree of visual space. For further details of the computational model see Dunbier et al. (2011).

We used a simplex search method to optimize the delay filter time constant of our model to provide the best fit to the observed response onset of CSTMD1 at 56°.s^−1^ averaged across 20 trials in a particularly healthy individual CSTMD1 recording. This time course was normalized to take account of receptive field inhomogeneity using the method described by Nordström et al. (2011). Briefly, this involved dividing the binned spike rate (bin size 20 ms) for targets commencing motion within the receptive field by the spline-smoothed and fully facilitated response along the same trajectory on a longer path commencing at the screen base (i.e., for a minimum of 500 ms before entering the same location within the receptive field).

### Velocity tuning

We determined velocity tuning using a test target (~1.1° square) that commenced upwards motion within the receptive field in the excitatory region just outside receptive field “hotspot” at one of twelve velocities logarithmically spaced between 5 and 200°.s^−1^. These test responses were analyzed within a short window (100 ms) commencing 50 ms after target onset to account for the absolute latency of the response. To partially account for inhomogenous receptive field shape (given that high velocity stimuli moving over a different range of locations in the same time analysis period) we varied test location across five different vertical trajectories (each horizontally separated by 3°) and two test trajectory origins (vertically spaced 5° apart). Test stimuli were either preceded by: (1) an adapting blank screen of mean luminance, allowing us to determine the “un-facilitated” velocity tuning; or (2) a facilitating stimulus consisting of a relatively low velocity (33°.s^−1^) target that drifted upwards from the bottom of the screen to the test location before accelerating. This permitted determination of velocity tuning in the facilitated state.

## Results

### Response reduction to stimuli on discontinuous paths

Targets drifted along prolonged single trajectories within the receptive field of CSTMD1 elicit a response that slowly builds to a “facilitated” level (Nordström et al., 2011). Does this facilitation build by successive stimulation of local regions along a continuous trajectory or is it established and maintained by global activity of CSTMD1, irrespective of the locality of the target within the receptive field? If the mechanism of facilitation is global, it ought to transfer to a new location. We tested this by designing a stimulus protocol where targets moved upwards though a sub-region of the receptive field, with four different degrees of spatial discontinuity (Figure 1). In each case targets moved at 56°.s^−1^, matched to the velocity optimum observed in our previous experiments (Geurten et al., 2007). Figure 1A illustrates one of the five sets of upward scans. Each of the four stimulus conditions had an equivalent amount of total motion energy, so varied only in the local distribution of this motion over time. As can be seen from the raw responses (Figure 1B), disruption of the path into smaller (shorter duration) segments leads to a strong reduction in the response, despite even the smallest segments still being 7° and thus representing local trajectories that must cross numerous underlying local motion detectors.

The reduction in response for short paths evident in Figure 1B is biased by the inhomogeneity of the receptive field, since the longest path illustrated (left) passes through the most sensitive part of the receptive field. However, after repetition of all five sets of segmented trajectories, the target has traversed the same total area of this inhomogeneous receptive field. We therefore reconstructed receptive fields (Figure 1C) by excising and concatenating the spiking responses that correspond to target traversal of a particular spatial region of the visual field.

These receptive fields were then averaged over several neurons (*n* = 7). As observed in Nordström et al. (2011), single segment paths reveal time courses building to a facilitated level over several hundred milliseconds (Figure 1C). Following this, CSTMD1's response then reflects the spatial structure of the receptive field, including a “hotspot” in the upper left corner of the region of interest. However, as target paths are split into smaller and smaller segments (left to right), the reconstructed receptive fields exhibit consistently weaker activity at all locations. Thus local spatial discontinuities cause responses to reset and begin to re-facilitate. The shortest path lengths are still long in comparison to the distribution of photoreceptors, given an interommatidial angle of just 0.5–1° within this part of the visual field in *Hemicordulia* (Horridge, 1978). This decrease in activity cannot, therefore, simply be attributed to the interruption of stimuli within the receptive field of single underlying ESTMDs. We conclude that the facilitated response state does not transfer to a new location and is unlikely to be due to a simple global mechanism.

### Quantitative analysis of the influence of path discontinuity

To quantify the reduction in activity due to local discontinuities, we calculated mean spike rate over each of the reconstructed receptive fields for CSTMD1 and plotted this against segment length (Figure 2A). Data for CTSMD1 (*n* = 7, mean ± SEM) are well fitted by a saturating exponential curve (*r*^2^ = 0.86) showing that responses are not fully saturated even at a path length of 56°. Hence, targets must traverse many dozens of ommatidia in order to produce a maximal response.

While our primary aim was to record from CSTMD1, we also recorded several times from a previously unidentified size-selective STMD neuron (1–2°), which we hereafter refer to as the “binocular small target motion detector 1,” BSTMD1. This provided an opportunity to repeat these experiments and thus test whether local facilitation was unique to CSTMD1 or also seen in other STMDs (and thus likely to be due to a mechanism expressed in the local inputs to these neurons). Because this neuron was previously unidentified, we subsequently characterized its receptive field and reconstructed its morphology following intracellular Lucifer Yellow injection (see *BSTMD1 Physiology and Neuroanatomy*, below). Local facilitation is not unique to CSTMD1, as BSTMD1 produces a similar curve (*n* = 2, Figure 2B). Thus, either local facilitation is a property of underlying processing elements common to the two neurons, or is simply a characteristic shared by the larger receptive field STMD neurons.

### Does facilitation depend on path length, path duration or velocity?

**Figure 3:**
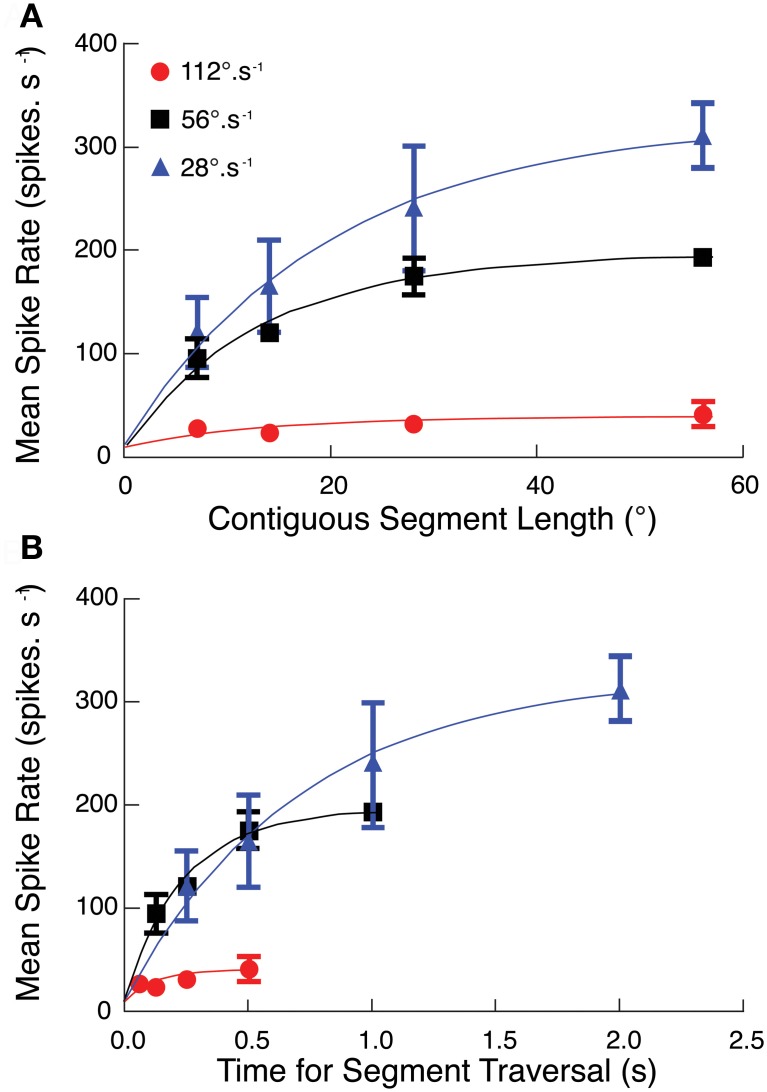
**(A)** The segmented path experiment (Figures 2, 3) at target speeds of 56°.s^−1^ is repeated at two additional velocities (28°.s^−1^, 112°.s^−1^). At all three velocities, CSTMD1 activity increases as segment length increases, with strongest responses at the slowest speed of 28°.s^−1^ (mean ± SEM, *n* = 6, three trials in 2 neurons). **(B)** As velocity increases, targets traverse the same number of ommatidia but for a shorter time. The effect of this on CSTMD1 activity is revealed by re-plotting data in **(A)** as a function of segment duration, rather than segment length. For a particular duration, CSTMD1 activity is similar whether the target moves at 28°.s^−1^ or 56°.s^−1^. For example, a target moving at 28°.s^−1^ over 14° (0.5 s) results in the same mean CSTMD1 spike rate as a target moving at 56°.s^−1^ over 28° (0.5 s).

To test whether facilitation depends on path length (i.e., the number of ommatidia traversed by a target) or the duration of the trajectory, we repeated the discontinuous path experiment at half and double the original velocity (i.e., at 28 and 112°.s^−1^). These speeds lie either side of CSTMD1's optimal velocity as suggested by our earlier work (Geurten et al., 2007). The different velocities stimulate the same spatial region with the same path lengths, but for different periods of activation. We found that mean spike rate over each of the reconstructed receptive fields was substantially stronger at 28°.s^−1^ than at the velocity tuning peak of 56°.s^−1^ expected from the Geurten et al. (2007) data (Figure 3A). We re-plotted this same data, but now as a function of the time for segment traversal, rather than the segment length (Figure 3B). Response dependence on segment duration is similar at both 28 and 56°.s^−1^ although the initial response (in the first 200 ms) would appear to be strongest for 56°.s^−1^.

The difference in velocity tuning highlighted by our experiments compared with Geurten et al. (2007) illustrates an inherent problem in defining the velocity tuning for a neuron that responds only to discrete features, yet has an inhomogeneous receptive field. Geurten et al. (2007) evaluated their responses in an “early” time window within a few hundred milliseconds after target motion commenced within the receptive field. This allowed them to estimate responses up to relatively high speeds without the target moving out of the receptive field. However it is clear from our data that such a stimulus does not allow facilitation to fully build for slower moving targets. Our data show that a target travelling at lower speeds over a long path may eventually reach a higher firing rate.

**Figure 4:**
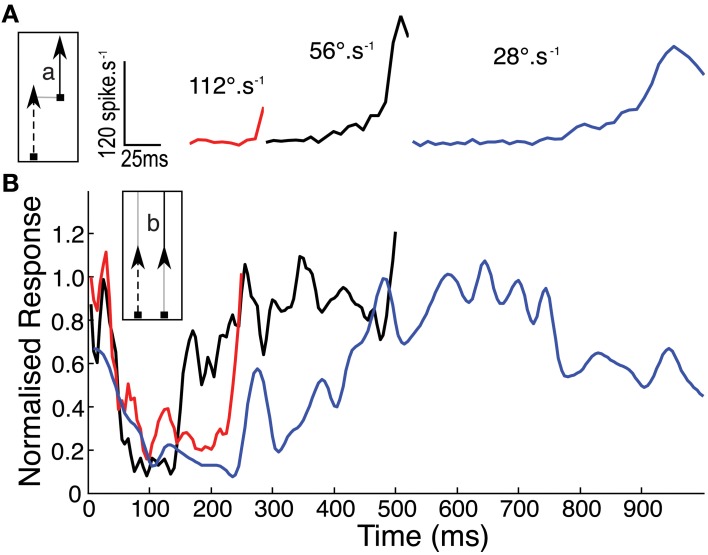
**(A)** Mean CSTMD1 response during the second segment (a) of the 28° two path stimulus, illustrates the onset time course at each of the three velocities (*n* = 30, 3 trials of 5 traversals in 2 neurons). **(B)** To account for spatial inhomogeneity of the receptive field, time courses in **(A)** are normalized by response during the second half (b) of the corresponding single path experiment (i.e., in the facilitated state). Normalized responses initially decrease as “left over” excitation from the first path decays. At 28°.s^−1^, response builds over hundreds of milliseconds more slowly than the 56 and 112°.s^−1^ time courses.

It is possible that our new data are influenced strongly by the inhomogeneity of the receptive field, since they always drift upwards through it (i.e., toward the more sensitive receptive field center). Hence the raw time course of the response at any velocity (Figure 4A) reflects both the receptive field structure and the underlying response kinetics. To account for this we normalized the second half of the 2-segment paths (Figure 1C) by dividing it through by the single path data. The assumption here is that the response is fully facilitated after the first 500 ms in the longest path (at 56°.s^−1^) such that subsequent response modulation is due primarily to the receptive field shape. The resulting time course can then be normalized by its own maximum to allow comparison of the response time course at different velocities, whilst ignoring the underlying spatial inhomogeneity (Figure 4B). This analysis reveals a clear dependence of time-course on target speed, with the slowest (28°.s^−1^) target producing a much slower roll-on in response than at 56°.s^−1^ or 112°.s^−1^. We should note that the final normalization may underestimate the speed of saturation in the fastest case, because the target never reaches a steady state before it leaves the receptive field (and indeed our stimulus display).

### Mechanisms underlying slow response roll-on

A simple explanation for the slow response onset at very low target speeds would be a long time constant in any filters either on the inputs to motion detectors or on their outputs. Such sluggish response kinetics might enable responses to accumulate as targets traverse successive local motion detectors. Nordström et al. (2011) discounted simple sluggish filter kinetics as an explanation for slow facilitation in CSTMD1. They showed that when targets stop within the receptive field, the responses decay back to resting levels in 1/10 of the time that they take to facilitate. However, such asymmetry between response onset and response decay could be explained if it was the delay filter intrinsic to local motion detection itself that had the long time constant (Hassenstein and Reichardt, 1956).

**Figure 5:**
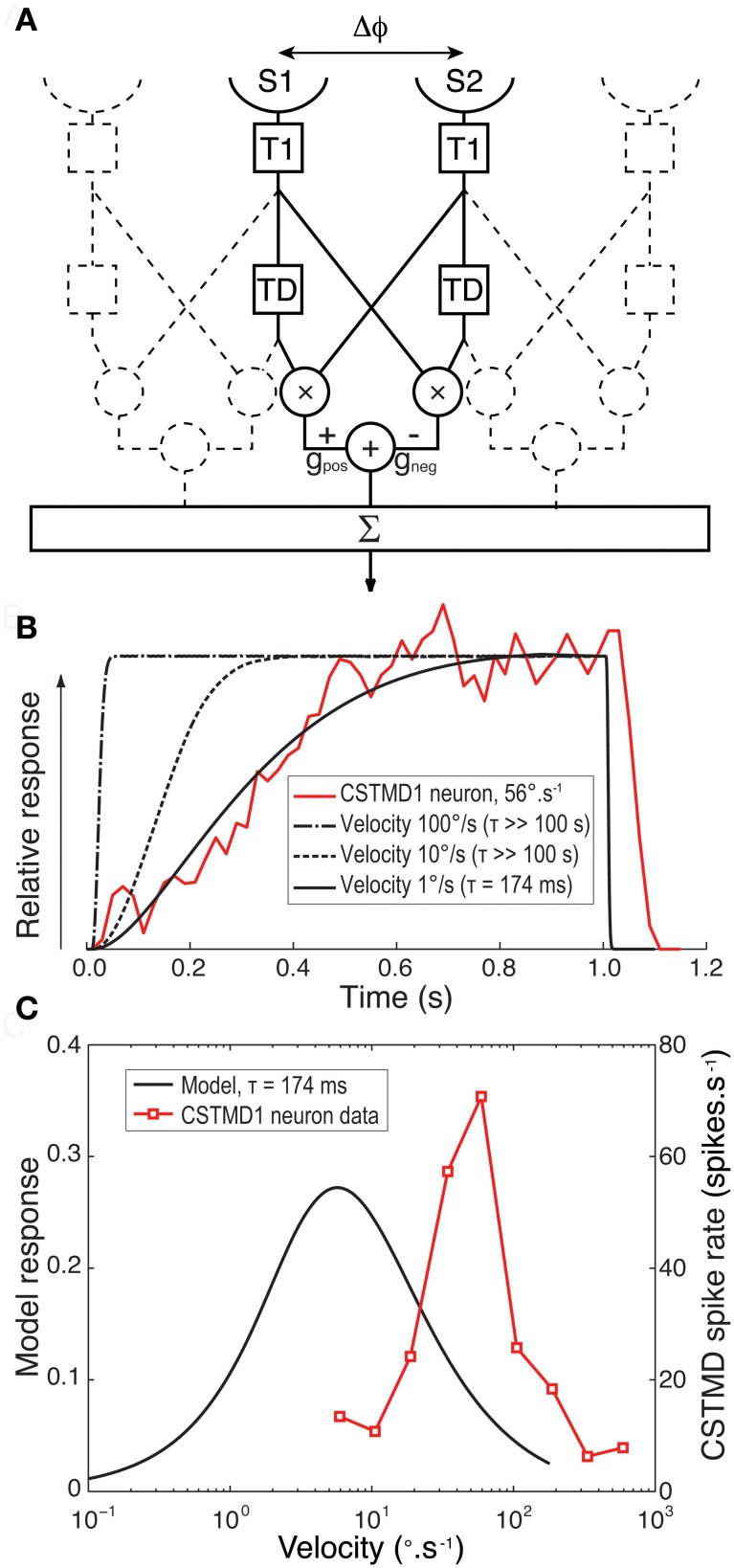
**(A)** A block diagram for our computational model for an EMD array. For each EMD in the array two adjacent sensors (S1 and S2) with separation Δϕ receive a luminance signal that is Gaussian blurred to represent the optics of the compound eye. Each signal arm is then convolved with a lognormal temporal filter (T1) that mimics the temporal properties of the insect photoreceptor. For a target moving left to right the output of S1 is then further convolved with an exponential delay filter (TD). The undelayed signal from S2 was multiplied by the delayed signal from the neighboring arm. This process was repeated in a mirror symmetrical fashion and the two outputs subtracted from each other with equal gain (G) to give an individual EMD's response. Responses were then summed across the array traversed by the target. **(B).** Spatially normalized CSTMD1 response to a target commencing motion within the receptive field at at 56°.s^−1^ reveals a time course that builds over hundreds of milliseconds (red line). The model EMD array was then fitted to this data with a delay time constant optimized to match this physiological response. An excellent fit (black line) is possible when modeling the response to a low target speed (1 °.s^−1^) with a long time constant (τ = 174 ms) for the delay filter (TD). However at faster target velocities (dashed lines) the model cannot fit the data and the model response rolls on too fast for even the largest time constants fitted (>100 s) **(C)** Velocity tuning of the EMD model with a time constant of 174 ms (black line) has a peak response at a speed 1/10th of that we previously observed from CSTMD1 (red line, Geurten et al., 2007).

Is it possible to reproduce a slow onset time course (as seen in CSTMD1) with slow delay time constants in primary motion detecting sub-units? To test this hypothesis we created a computational model of a 1-dimensional array of motion detectors based around known properties of the insect eye (Figure 5A). We avoided our predictions being confounded by speculation over the complex adaptive filters and additional nonlinearity required to explain the selectivity for small targets (Wiederman et al., 2008, 2010) by modeling only elementary motion detectors (EMDs) of the correlation type. Although not an STMD computational model *per se*, we previously showed that responses summed across such EMDs arrays provide a good explanation for velocity tuning to features seen in CSTMD1 and may even explain the dependence of the latter on target dimensions along the direction of travel (Geurten et al., 2007). We used a simplex search method to optimize the delay filter time constant of our model to provide the best fit to the observed response of CSTMD1 at 56°.s^−1^ (redline in Figure 5B). We repeated this optimization for a number of different target speeds, from 1 to 100**°**.s^−1^.

At very low speeds (e.g., 1°.s^−1^) our EMD model can provide an excellent fit to the observed CSTMD1 time course with a long delay constant (τ = 174 ms, Figure 5B, solid black line). Consistent with the above hypothesis, the EMD response is also strongly asymmetric, showing slow onset and rapid offset once target motion ceases. By even 10**°**.s^−1^, however, the model time course is far too short to explain the observed data, even if we increase the delay time constant to the maximum constraint imposed by our optimization (τ >100 s). By speeds comparable to those used in the actual experiment, the model response onset is very rapid, dominated primarily by the kinetics of the early visual processing rather than that of the long time constant delay filter. This is because a target moves into and out of the receptive field of the EMD inputs in a much shorter time than the time constant of the delay filter. It would thus appear that the response of an array of EMDs to targets moving at biologically relevant velocities cannot reproduce the slow onset in CSTMD1s response under any conditions.

Furthermore, given the long time constant delay required to fit the observed responses for even very low speed targets, such a motion detector could not explain the relatively high sensitivity of CSTMD1 to higher speed motion, since it would be tuned to correspondingly slow speeds (Borst and Bahde, 1986). This is illustrated in Figure 5C, which compares the velocity tuning (in the steady state) for the EMD model with a slow delay time constant (τ = 174 ms) with that of CSTMD1 (redrawn from Geurten et al., 2007). The model velocity tuning peaks at velocities less than 1/10^th^ of those shown to be the best drivers for CSTMD1, either in the earlier work (Geurten et al., 2007) or as revealed by our analysis above (Figure 4).

### Could facilitation boost responses to low velocity features?

From our analysis above, we conclude that the characteristic features of response facilitation cannot be explained by asymmetries in response time course that result from the basic mechanisms involved in local motion detection. But we also nevertheless observe dependence in the response time course on the velocity of target motion. Could this be due to facilitation primarily operating to boost responses to lower velocities over a prolonged time course? To test this we examined the influence of facilitation to a relatively low velocity target on subsequent velocity tuning across a range of target speeds using a modification of the stimulus protocol of Geurten et al. (2007). Our stimulus used a test path commencing within the receptive field. In two sets of stimuli this was either preceded by an adapting blank screen of mean luminance, allowing us to determine the “un-facilitated” velocity tuning in a short time window after stimulus onset, or by a relatively low velocity (33°.s^−1^) target that drifted upwards to the same location to allow us to measure the velocity tuning in the same time window but in the facilitated state.

**Figure 6:**
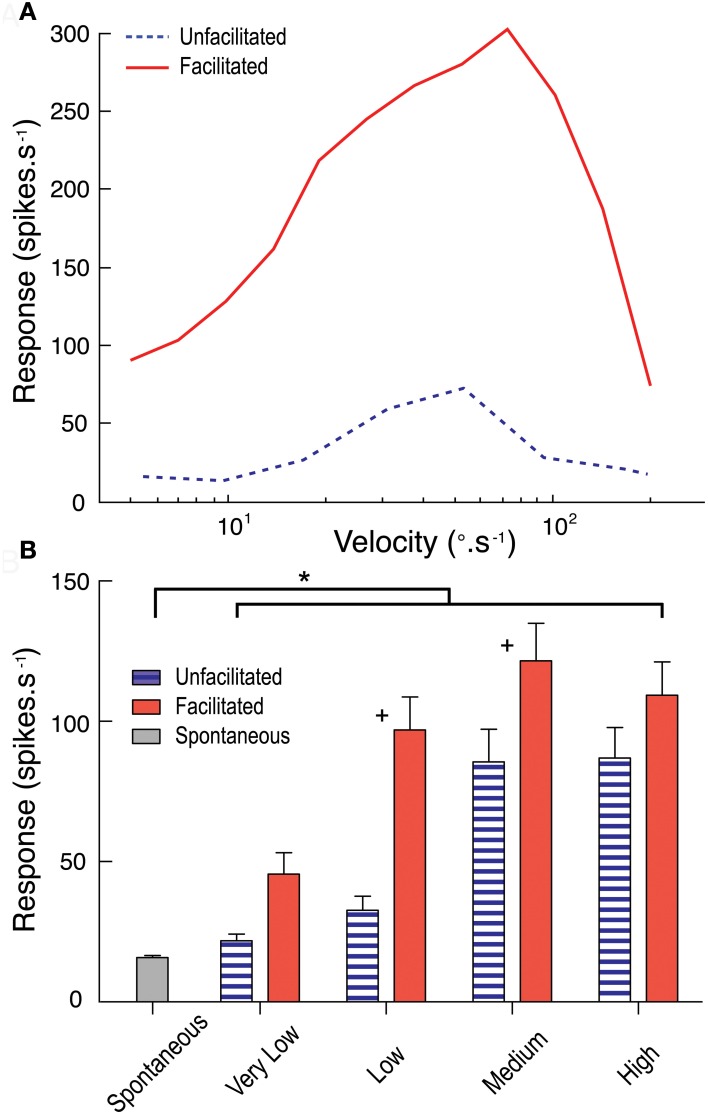
**(A)** The unfacilitated velocity tuning curve (blue dotted line) measures CSTMD1's response to targets commencing movement within the receptive field. The facilitated velocity tuning curve (red line) is measured at the equivalent receptive field location, however follows a facilitating stimulus (target moving at 33°.s^−1^). Facilitated responses are much stronger across the velocity range. **(B)** Results pooled by grouping velocity ranges (very low, < 10°.s^−1^; low, between 10 and 30°.s^−1^; medium, between 30 and 100°.s^−1^; and high, > 100°.s^−1^; 10 trials per velocity in 3 neurons) show facilitation has the largest effect at low and medium range velocities (+ = significant difference between facilitated and unfacilitated, *P* < 0.05; ^*^= significant difference from spontaneous activity, *P* < 0.05).

The results are shown in Figure 6. Following the slow facilitating stimulus, CSTMD1 is much more responsive to targets for a range of subsequent velocities (Figure 6A). We should note that due to the confounding influence of receptive field shape (and high velocity stimuli moving over a different range of locations in the same time period) the specific shape of such curves is quite variable, depending on the site selected for the test stimulus (data for a single neuron at 1 location shown in Figure 6A). We therefore varied test location across several positions within the receptive field and pooled data from from velocity ranges (very low, < 10°.s^−1^; low, between 10°.s^−1^ and 30°.s^−1^; medium, between 30°.s^−1^ and 100°.s^−1^; and high, > 100°.s^−1^) across three CSTMD1 recordings (Figure 6B). The largest and most significant boost to subsequent responses by the facilitating stimulus is for slower target velocities. Indeed, these results show that responses to velocities below the un-facilitated optimum (particularly between 10 to 30°.s^−1^) are the most enhanced by facilitation.

### BSTMD1 physiology and neuroanatomy

The second neuron included in this study, BSTMD1, has not previously been described in the literature. To assist future researchers in identifying their recordings should they encounter similar neurons, we therefore reconstructed its receptive field in detail, and its morphology following intracellular injection of Lucifer Yellow, using standard methods (as described in Geurten et al., 2007).

**Figure 7:**
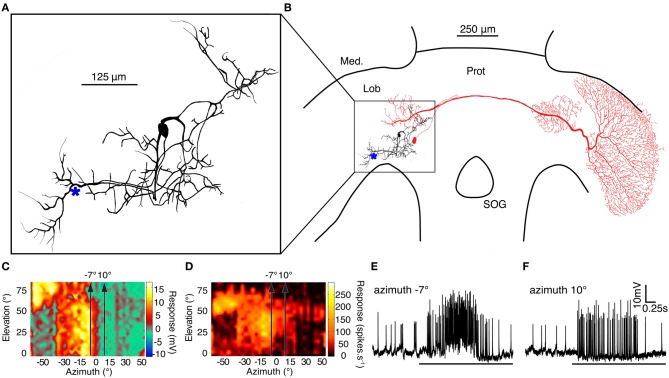
**(A)** A Lucifer Yellow CH fill of the compact mid-brain small target motion detector neuron, BSTMD1. (^*^ indicates approximate recording site) **(B)** BSTMD1 (black) in context of the dragonfly brain showing a likely input arborisation in the proximal lobula and a second region of inputs/outputs in the lateral midbrain. CSTMD1s (red) inputs are in a similar region of the lateral midbrain. **(C)** The graded (generator potential) receptive field of BSTMD1 mapped by upward motion of a 1.2° square target. The depolarization response is monocular and ceases at the midline whereas the contralateral visual hemifield is hyperpolarized. However, the spiking receptive field **(D)** is prominently binocular, with spikes on both graded responses. (**E** and **F**) Example traces from targets moving upward through the receptive field at the two positions indicated by arrows in receptive field plots (**C** and **D**) at −7° and 10° azimuth.

BSTMD1 is a compact, multipolar mid-brain intrinsic neuron, with a putative input arborization on the proximal (output) side of the lobula and with several other regions of inputs or outputs in the lateral midbrain (Figures 7A,B). BSTMD1 has a pronounced binocular receptive field and gives mixed mode responses, with both action potentials and large graded components (Figures 7C–F). Responses are not direction selective. Figures 7E,F shows raw responses to targets drifted upwards through the receptive field, a few degrees either side of the frontal midline (along the stimulus tracks shown in Figures 7C,D). BSTMD1 spikes in response to targets presented in either visual hemifield, but intriguingly exhibits graded depolarizing responses only to stimuli presented in the ipsilateral hemifield (Figures 7E,F). Such stimuli elicit large graded depolarizations (up to 10mV), suggesting that our recording site (indicated approximately by the ^*^ in Figure 7A) is very close to the ipsilateral excitatory synaptic input. Contralateral stimuli elicit more biphasic spikes (Figure 7F) of larger amplitude. These rides upon a pronounced hyperpolarization at our recording site, suggesting that the contralateral excitatory inputs are actually more remote (presumably in the dendrites located in the lateral mid-brain) and that action potentials may be initiated at more than one location.

The midbrain dendritic region of BSTMD1 corresponds well with the position of the midbrain arborisations of CSTMD1 (illustrated in red in Figure 2B). In our earlier work on CSTMD1 (Geurten et al., 2007) we noted that these two mid-brain arborisations are mirror symmetric. Hence it is possible that CSTMD1 is pre-synaptic to BSTMD1 and is responsible for its contralateral excitatory input. It is also possible that BSTMD1 in turn makes bi-directional synaptic contact with CSTMD1 and is responsible for its ipsilateral input. This arrangement is further suggested by the very sharp boundary between the depolarization and hypolarisation sub-regions of the receptive field (Figure 7C), which closely correspond to an equally sharp boundary in the CSTMD1 excitatory field. This boundary is surprising for either neuron when we consider that at elevations of 40–50° this represents not only the dorsal acute zone (an area of unusually large ommatidial facets) but also to a region of approximately 15° of binocular overlap in the underlying ommatidial input (Horridge, 1978). An obvious potential role for such binocularity would be the summation of local motion detector responses to improve reliability for discriminating small features in this highly salient part of the visual world, and this is certainly supported by the characteristic hotspot in the CSTMD1 receptive field (evident at the upper left corner of receptive fields in Figure 1C; see also Geurten et al., 2007). The physiology of BSTMD1, however, suggests that ipsilateral excitation only comes from local inputs up to the midline itself. Local motion detectors from the binocular zone of the contralateral eye could still contribute to the sensitive excitatory “hotspot.” This would, however, require additional local neurons to cross the brain and connect directly with the neurons at the corresponding receptive field location in the contralateral hemisphere of the lobula or brain. Such neurons have yet to be identified.

## Discussion

We have established that the slow build up in response that characterizes facilitation of CSTMD1 (and most likely other higher-order wide-field STMD neurons such as our newly described BSTMD1) is re-set to a “naive” time course after relatively small lateral displacements (6°) in the target path. This reduces the overall activity of the neuron in response to discontinuous target motion. We further ruled out the possibility that the slow response time course is simply a by-product of a long neural delay in correlation mechanism underlying local motion detection. Together, our findings discount global properties of the neuron, such as axonal integration of its inputs or active conductances within the axon, as potential mechanisms for facilitation. The lack of transfer to new locations for discontinuous motion, combined with our observation that prior facilitation by targets moving slowly along a prolonged continuous path exert the most potent effect on subsequent stimuli that also move at low velocity (Figure 7), suggests that facilitation does not spread instantaneously to new locations within the receptive field, but rather spreads slowly away from the current target location.

The next challenge for future work will be to quantify the extent of spread of facilitation in space, time and direction away from the current location of the target. Full characterization of these parameters should provide better clues as to the underlying mechanisms and possible pharmacological targets for experimental testing. In approaching these experiments, it is also worth considering the degree to which facilitation is a bottom-up process, i.e., an emergent property of the underlying network of neurons, versus the possibility that it is also recruited by a top-down modulation of stimulus salience. Recent findings have suggested that responses of visual neurons are strongly modulated by the behavioral state of the animal during the recording (Chiappe et al., 2010; Maimon et al., 2010; Jung et al., 2011). It is worth remembering that in our experiments the animal is restrained with wax and subjected to long periods of repetitive stimulation—hardly a natural condition. Certainly we observe a degree of pronounced habitation in response to repeated stimulation of these neurons by identical stimuli (Geurten et al., 2007; Bolzon et al., 2009). control against this via randomization of experiment order and with long rest breaks between trials. While at this stage it is unrealistic to propose recording from CSTMD1 in unrestrained or tethered flight, it ought to at least be possible to test for similar modulation as shown in other insect visual neurons by exogenous application of neuromodulator agonists. In particular, the Octopamine agonist chlordimeform (CDM) has recently been shown to mimic the effect of free flight in altering the responses of wide-field motion sensitive neurons in flies (Longden and Krapp, 2010; Haan et al., 2012).

Whatever the underlying mechanism, the facilitation we observe must to some degree represent a form of second order motion processing. STMDs respond to relatively high velocities (indicating short neural delays in underlying local motion detectors) and show very sharp tuning to very small features on the scale of single ommatidia. This tells us that the primary motion detectors must be operating on short time scales and at the resolution limits of the eye. Facilitation, on the other hand, is a non-linearity that apparently operates across spans of tens of ommatidia even for optimum speed targets, and over time-courses of hundreds of milliseconds. A possible consequence of such a second-order non-linearity, cascaded with the (already highly nonlinear) operation of local target motion detection (Wiederman et al., 2008; Wiederman and O'Carroll, 2011) would be a potential sensitivity to non-Fourier motion stimuli such as theta motion (Zanker, 1994). While we have not yet subjected CSTMD1 to non-Fourier stimuli, their application to freely flying *Drosophila* reveals sensitivity to second order motion (Theobald et al., 2008). This sensitivity also develops over a prolonged time course (several hundred milliseconds) compared with the response to Fourier motion (Theobald et al., 2008; Lee and Nordström, 2012).

While our future work may test these hypotheses and reveal the underlying mechanisms in more detail, the properties that we have revealed to date beg the question as to what role facilitation plays in the behavior of the animals? The relatively slow build up, in some cases over half a second or more, seems an eternity compared with the minimum response delays observed in dragonflies to target stimuli during prey pursuit, which are only 25–30 ms (Olberg et al., 2000). Given this short latency, it seems unlikely that facilitation is necessary for target detection *per se*—at least if targets are viewed under ideal conditions (i.e., optimal size, speed, and high contrast against the background). One possibility is that once a target is initially detected by the underlying network of local motion detectors, localized facilitation helps maintain an elevated sensitivity in the neurons adjacent to the most recently “seen” location, providing a form of robustness against possible future occlusions (e.g., as the target passes in front of a luminance matched feature of the background scene). While flights for pursuit and capture of prey can be very brief in total duration in dragonflies (Olberg et al., 2000, 2005), we have frequently observed males of *Hemicordulia* and a number of similar perching and hawking dragonfly species engage in prolonged pursuit flights of conspecifics, lasting several seconds or longer. The tight turns so characteristic of insect territorial pursuit flights (Collett and Land, 1975, 1978) would frequently place their target against complex background texture. Furthermore, while retinal velocities of the background scene would be extremely high (Voss and Zeil, 1998) if such pursuit flights are to be effective, the relative slip speed for the target would be inherently much slower. Hence even a slow mechanism such as we observe could serve a useful role in boosting the relative “salience” of the current target location, and aid in target re-acquisition following either temporary occlusion by foreground features or loss against the background.

### Conflict of interest statement

The authors declare that the research was conducted in the absence of any commercial or financial relationships that could be construed as a potential conflict of interest.

## Abbreviations

BSTMD1: Binocular small target motion detector 1
CSTMD1: centrifugal small target motion detector 1
TSDN: target selective descending neuron
STMD: small target motion detector
ESTMD: elementary small target motion detector
EMD: elementary motion detector
LPTC: lobula plate tangential cell
ROI: region of interest.
